# Relationship between Canadian medical school student career interest in emergency medicine and postgraduate training disposition

**Published:** 2017-06-30

**Authors:** Riyad B. Abu-Laban, Ian M. Scott, Margot C. Gowans

**Affiliations:** 1 Department of Emergency Medicine, University of British Columbia, British Columbia, Canada; 2 Department of Family Practice, University of British Columbia, British Columbia, Canada

## Abstract

**Background:**

Canada has two independent routes of emergency medicine (EM) training and certification. This unique situation may encourage medical students with EM career aspirations to apply to family medicine (FM) residencies to subsequently acquire College of Family Physicians of Canada (CFPC) training and certification in EM. We sought answers to the following: 1) Are medical students who indicate EM as their top career choice on medical school entry, and then complete a FM residency, more likely to undertake subsequent CFPC-EM training than other FM residents who did not indicate EM as their top career choice; and 2) What are the characteristics of medical students in four predefined groups, based upon their early interest in EM as a career and ultimate postgraduate training disposition.

**Methods:**

Data were accessed from a survey of medical students in 11 medical school classes from eight Canadian universities and anonymously linked to information from the Canadian Residency Matching Service between 2006 and 2009.

**Results:**

Of 1036 participants, 63 (6.1%) named EM as their top career choice on medical school entry. Of these, 10 ultimately matched to a Royal College of Physicians and Surgeons of Canada (RCPSC) EM residency program, and 24 matched to a FM residency program, nine of whom went on to do a one-year CFPC-EM residency program in contrast to 57 of the remaining 356 students matching to FM residency programs who did not indicate EM was their top career choice (37.5% vs 16.0%, p=0.007). Statistically significant attitudinal differences related to the presence or absence of EM career interest on medical school entry were found.

**Conclusion:**

Considering those who complete CFPC-EM training, a greater proportion indicate on admission to medical school that EM is their top career choice compared to those who do not. Moreover, students with an early career interest in EM are similar for several attitudinal factors independent of their ultimate postgraduate training disposition. Given the current issues and challenges facing FM and EM, these findings have implications that merit consideration by both the CFPC and the RCPSC.

## Introduction

Canada is unique in having two routes of emergency medicine (EM) training and certification overseen by two independent colleges. The Royal College of Physicians and Surgeons of Canada (RCPSC) offers a five year program leading to specialist certification in EM.^1^ The College of Family Physicians of Canada (CFPC) offers a one-year program, with an entry prerequisite of completion of a two year Family Medicine (FM) Residency, leading to a certificate of special competence in EM.^2^ Although the stated goals of these two programs differ, the majority of CFPC-EM graduates practice full-time EM in medium and large centres, often side-by-side with RCPSC graduates, rather than in the blended FM and EM practice the program was intended and designed to enable.^3^–^5^ Graduates of each program are also active across the spectrum of Canadian EM administration and academia.^3^

This approach to Canadian EM training and certification has generated debate for decades,^6^–^8^ and in 2011 the Canadian Association of Emergency Physicians (CAEP) released a statement advocating for a unified and coordinated approach to train both clinical and academic emergency physicians.^9^,^10^ More recently, in 2016, the final report of the “Collaborative Working Group on the Future of EM in Canada” was released. This working group, made up of members from the CFPC, RCPSC and CAEP, found that a considerable health human resource shortfall in Emergency Physicians (EPs) exists nationally and is expected to grow, and made a number of recommendations intended to improve EM training and inter-college collaborations in this area.^3^

While issues of standards and the importance of matching EM and FM training resources to career disposition are important, we sought to focus on how the current situation may encourage those with EM career aspirations to apply to FM residencies with the goal of practicing full time emergency medicine through a training route not intended or designed for this career path.^11^ This suggestion has never been rigorously investigated, and if true has important implications for both EM and FM and Canada as a whole where about 10% of CFPC graduates undertake CFPC-EM certification.

Most of the research on the evolution of medical student career aspirations has been performed in the United States.^12^–^21^ It is known that student’s demographic, experiential, and attitudinal characteristics upon medical school entry are predictive of their ultimate career path, and that the stability of early medical school career aspirations varies with the field involved.^22^,^23^ One study found that EM was both the most popular and the most stable choice among all specialties.^13^ Another found Canadian medical students with a career interest in EM have attributes that differentiate them from those with an interest in FM, the surgical specialties, and the medical specialties.^24^ However the evolution of the EM career aspirations of Canadian medical students, characteristics associated with FM and EM training choices, and influence of Canada’s dual college dual certification EM system on student’s pursuit of postgraduate training, have to date been poorly understood.^25^

We therefore designed this study to answer the following questions:

1. Are medical students who indicate EM as their top career choice on medical school entry and then complete a FM residency more likely to undertake subsequent CFPC-EM training than other FM residents who did not indicate EM as their top choice (primary outcome); and
2. What are the characteristics of medical students in four predefined groups, based upon their early interest in EM as a career and ultimate postgraduate training disposition (secondary outcomes).

## Methods

Data were accessed from a survey administered between 2002 and 2004 to medical students during their first two weeks of medical school in 11 medical school classes from eight Canadian universities in British Columbia, Alberta, and Ontario and anonymously linked to information from the Canadian Residency Matching Service for the years 2006 through 2009.^23^,^24^,^26^ All students in medical school at the study universities during the first two weeks of their first year were eligible. Our methodology involved the administration, typically after a large-group lecture, of a 41-item written survey that took approximately 15 minutes to complete. The survey asked students to rank their top three career choices from the following options: Emergency Medicine, Urban Family Medicine, Rural Family Medicine, Internal Medicine, Obstetrics and Gynecology, Pediatrics, Psychiatry, Surgery, “other,” and “do not have a preference.” Using a 5-point Likert scale, students were then asked to indicate the extent to which their career interest was influenced by each of 27 attitudinal items. Two years after their graduation, survey data from participating students were confidentially linked with the postgraduate training disposition (residency information) and deidentified. Our focus was on a comparative evaluation of the ultimate postgraduate training disposition of students at the study universities who indicated EM was their top career choice in Canada’s 2006 and 2007 medical school graduation years.

SPSS (Version 20, PC) was used for the analysis. Descriptive statistics were generated and the *Chi* Square test was used for the primary analysis comparing proportions of students applying for CFPC-EM training who indicated they had a career interest in emergency medicine and all other FM residents. A power calculation was not performed *a priori*, as our sample size was predetermined by the number of participants in the database from the original study.

For the secondary analysis, demographic, experiential, and attitudinal characteristics of four groups of medical students were compared: 1) career interest in FM, undertook FM training; 2) career interest in FM, undertook CFPC-EM training; 3) career interest in EM, undertook RCPSC-EM training; and 4) career interest in EM, undertook CFPC-EM training. Associations between characteristics and career interest were identified using analysis of variance with least significant difference *post-hoc* test for continuous variables and cross-tabulation with the *Chi* Square test for categorical variables.

Factor analysis was performed to condense the 27 career influences into a smaller number of factors. Variables with an eigenvalue of >1.0 and a correlation of >0.5 were included in a factor. Analysis of variance with Scheffe’s *post-hoc* test was used to identify differences in the resulting factors according to career interest. A p-value less than or equal to 0.05 was deemed statistically significant. Approval to conduct this study was obtained from the University of British Columbia research ethics board.

## Results

**Figure 1:**
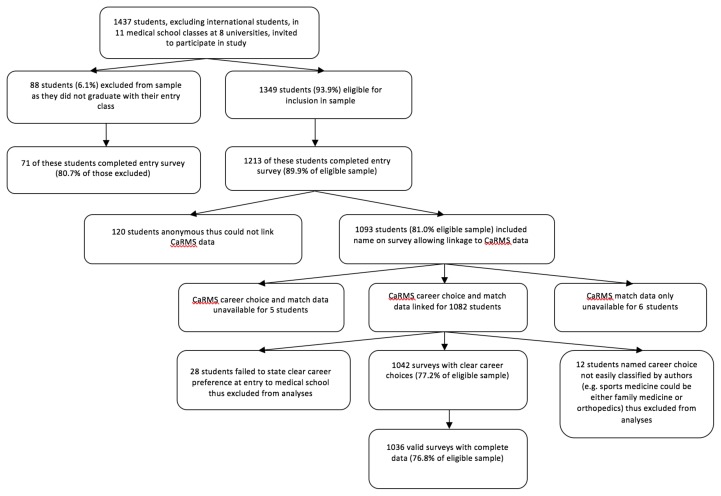
Origin of study population

Of 1349 eligible students, 1036 (76.8%) participated. Data were collected from three different medical school entry year cohorts comprising only two medical school graduation years (2006 <n=362> and 2007 <n=674>) due to variability in program length at participating schools. Figure 1 provides an illustration of the origin of the study population.

Of the eligible students, 63 (6.1%) indicated on medical school entry that EM was their top career choice. Of these 63, 10 (15.9%) ultimately applied for and eight (12.7%) matched to a RCPSC-EM residency program, and 21 (33.3%) applied for and 24 (38.1%) matched to an FM residency program. There were more students matching than applying to a FM residency because some students matched on the second round to a program they didn’t apply to on the first round of the match. Of the 380 medical students who matched to FM residency programs, 24 indicated EM was their top career choice on medical school entry. Nine of these 24 students (37.5%) went on to do a one-year CFPC-EM residency program in contrast to 57 of the remaining 356 students (16.0%) who went on to do a one-year CFPC-EM residency program but did not indicate EM was their top career choice on medical school entry (p=0.007).

Table 1 provides demographic and experiential characteristics of the four defined subgroups of the study population. No statistically significant differences among these groups were found. Table 2 provides attitudinal characteristics of these same groups. There were statistically significant differences between the groups regarding social orientation, hospital orientation, focus on non-urgent care, research interest, and length of postgraduate training.

## Discussion

Our results indicate a substantially and statistically significantly greater proportion of students who indicated EM was their first career choice on admission to medical school, matched to a FM residency, and then subsequently pursued CFPC-EM training compared to the proportion of students who did not indicate EM was their first career choice on admission to medical school, matched to a FM residency, and then subsequently pursued CFPC-EM training (37.5% vs 16.0%, p=0.007). In addition, our results indicate that students with an early career interest in EM are similar on three of five attitudinal factors independent of their ultimate postgraduate training disposition; those with an early career interest in FM who undertook CFPC-EM training or FM training had similar characteristics, and those with an early career interest in EM who undertook RCPSC-EM training or CFPC-EM training had similar characteristics.

These findings have direct relevance to issues currently facing both FM and EM, and by extension to a large proportion of Canadian physicians practicing in one or both of these fields.

Our findings that indicate that a proportion of CFPC-EM graduates have a career interest in EM early in medical school has relevance to the number of FM training slots that ultimately lead to FM practice. While it is beyond the scope of our study to discern the reason(s) for medical students with an initial interest in EM to be highly represented in the FM pool that subsequently applies for CFPC-EM training compared to all other FM residents, one or a combination of two explanations seems likely:

1. There is a primary care orientation among students interested in both FM and EM predisposing career path switching; and/or
2. Students with a career interest in EM at medical school entry know or become aware of an alternate and shorter EM training route through a FM residency and subsequent CCFP-EM certification, and pursue this either primarily or as a back-up for applications to RCPSC EM residency programs.

Regardless of its explanation, the phenomenon we observed has health human resource implications regarding the planning and implementation of training resources for both EM and FM, one of which may be a reduction in the capacity of CFPC-EM programs to meet the needs of students inclined towards rural FM enhanced practice that includes work in an emergency department. This suggestion is underscored by the fact that national surveys of EM residents from both training programs carried out in 2015 by the Collaborative Working Group on the Future of EM in Canada found most CFPC-EM residents anticipate they will do little if any FM during their clinical practice.^3^

While the raw numbers we report are small, they highlight statistically significant “switching behaviour” among medical students over the course of their education. It is known that the career aspirations of approximately 50% of medical students change over the course of their education, and that FM is the most stable career with 60% of medical students who indicated FM was their top choice on medical school entry ultimately matching to FM residency programs.^23^

The design and training duration of the two Canadian EM programs are markedly different,^1^,^2^ and may lead to unintended consequences related to student career choice. A 2011 study of the knowledge and attitudes of 406 medical students in Ontario led the authors to conclude “there is the potential that students interested in the FRCP training program are matching to FM programs with no intention of pursuing general practice.”^11^ Our finding that the proportion of students with an early career interest in EM who undertake CFPC-EM training is substantially higher than that of other FM residents who also end up taking CFPC-EM training but did not have an early career interest in EM provides additional evidence in support of this suggestion.

Since a 2003 US publication noted that interest in EM as a career is increasing,^27^ how demand for EM training programs is expected to evolve in Canada merits consideration. Our results should be considered in the wider context of the evidence and ongoing debate regarding whether or not changes to the Canadian system of EM training and certification are warranted. It is clear that the majority of Canadian emergency physicians, irrespective of which EM certification they hold, are dissatisfied with the status quo,^28^ and a 2010 CAEP national task force concluded “the existence of two training programs did not serve the current and future needs of the EM community, the specialty of EM, and the citizens of Canada.”^29^

It has been suggested that neither current Canadian EM program is both efficient and effective in meeting its stated goals, and that neither program is designed to produce full-time “clinical” emergency physicians.^6^,^7^,^30^ The reality of the career deployment of those undertaking CFPC-EM training led to specific recommendations by the Collaborative Working Group on the Future of Emergency Medicine in Canada to the Canadian College of Family Physicians on how best to optimize the CFPC-EM residency curriculum and approach.^3^ Our results provide further encouragement to both Colleges to evolve and improve their EM residency programs to better optimize the alignment of educational experience to trainee aspirations and career plans.

### Limitations

Our results should be interpreted with several limitations in mind. First, when our survey instrument was designed it was not anticipated that a categorization of career interest by the two EM training streams would be beneficial. As a result, it is uncertain that all students who indicated they had a career interest in EM upon commencing medical school were indeed expressing an interest in EM specialization through the RCPSC. It seems likely that most were, since options also existed to choose rural FM, urban FM, or a list of other specialties. Second, although our results suggest a significant proportion of students with an EM career interest use FM training as a route to this, our design and anonymous data linking precluded us from directly obtaining explanations from students for their behavior. For example, as we were unable to assess changes in career inclinations over the course of participant’s medical school training, it is conceivable that an increased awareness that EM can be a component of enhanced FM practice, particularly in rural settings, may have led some students with a primary interest in rural FM to pursue CFPC-EM training. Third, due to the relatively small proportion of students with an initial career interest in EM, and their categorization into 4 groups, the statistical power of our secondary outcome analysis was limited. However, despite the relatively small numbers, our *a priori* defined primary outcome comparison was both striking and statistically significant, suggesting it is likely that a true and relevant difference exists between the two groups. Fourth, our data describe the ultimate disposition of the 2006 and 2007 medical school classes at the study universities; for those undertaking a FM residency and then obtaining CCFP-EM certification, this would result in starting practice in 2010 or earlier. This time lag arose from the combined effects of several sequential and necessary steps including: 1) publication and dissemination of the primary analysis in 2011;^22^ 2) the discovery from the primary analysis that an unexpectedly high proportion of medical students switched from a stated career interest in Family Medicine to pursuing specialist Emergency Medicine training; 3) discussions and the resulting collaboration that led to the research questions we posed; 4) the complex linkages and permissions (both REB and CARMS) that were needed to access the required data; and 5) our analysis and interpretation of this data, and writing it up in the context of recent knowledge and the changes to EM training that both Colleges are undergoing. Although some changes have occurred since 2010, in particular the establishment of the Triple C approach to Family Medicine training, we are unaware of any compelling reason to suspect a more recent analysis would result in substantively different findings.^3^ Finally, we only surveyed eight of Canada’s medical schools, and did not include francophone schools, thus potentially limiting the generalizability of our results.

### Conclusion

Considering those who completed CFPC-EM training, a greater proportion had indicated on admissions to medical school many years earlier that EM was their first choice for a career in medicine (9/24 or 37.5%) compared to those who had not (57/356 or 16%). Moreover, students with an early career interest in EM are similar for several attitudinal factors independent of their ultimate postgraduate training disposition. These findings support the suggestion that the current dual college dual certification approach in Canadian EM may encourage medical students with EM career aspirations to apply to FM residencies with the goal of subsequently acquiring CFPC-EM certification and practicing full time EM through a route not intended or optimized for this role. These findings have implications that should be considered by both the CFPC and RCPSC.

## Figures and Tables

**Table 1:** Demographic and experiential characteristics of study population categorized by career path

	Career Path				
		
Top Career Choice on Medical School Entry	FM		EM		
		
Post-Graduate Training Disposition	FM	CFPC-EM	RCPSC-EM	CFPC-EM	
	n=160	n=20	n=8	n=9	

**Age at entering medical school** (years)	25.7	26.3	23.5	25.1	F = 0.72, p=0.544
**Gender** (% male)	31.2	42.9	50.0	55.6	*X*^2^ = 4.02, df=3, p=0.259
**Relationship status** (% single)	59.4	61.9	100.0	66.7	*X*^2^ = 5.42, df=3, p=0.143
**Parental Education** (% postgraduate university)	32.5	28.6	50.0	55.6	*X*^2^ = 3.22, df=3, p=0.359
**Family / friends in family medicine** (% yes)	23.1	33.3	25.0	55.6	*X*^2^ = 5.39, df=3, p=0.145
**Family/friends in any field of medicine** (%yes)	31.9	38.1	25.0	55.6	*X*^2^ = 2.62, df=3, p=0.454
**Home town population** (% <50,000)	35.0	42.9	12.5	22.2	*X*^2^ = 2.99, df=3, p=0.394
**Rural Childhood** (>50%)	30.6	38.1	25.0	22.2	*X*^2^ = 0.96, df=3, p=0.810
**Parents in rural community** (% yes)	35.0	33.3	12.5	22.2	*X*^2^ = 2.26, df=3, p=0.520
**Siblings in rural community** (% yes)	22.5	14.3	0.0	33.3	*X*^2^ = 3.70, df=3, p=0.295
**Grandparents in rural community** (% yes)	26.2	38.1	12.5	33.3	*X*^2^ = 2.37, df=3, p=0.499
**Proposed work community** (% < 50,000)	31.9	38.1	0.0	22.2	*X*^2^ = 4.48, df=3, p=0.214
**Prolonged time in developing nation** (% yes)	36.9	23.8	12.5	33.3	*X*^2^ = 3.19, df=3, p=0.364

**Table 2:** Attitudinal characteristics of study population categorized by career path

	Career Path				
		
Top Career Choice on Medical School Entry	FM		EM		
		
Post-Graduate Training Disposition	FM	CFPC-EM	RCPSC-EM	CFPC-EM	
	n=160	n=20	n=8	n=9	

**Factor 1 – Medical Lifestyle**	3.81	3.94	3.62	4.00	F = 0.44, df = 194, p=0.73
**Factor 2 – Social Orientation**	4.13^c,d^	4.06^c,d^	2.56^a,b^	2.83^a,b^	F = 24.61, df = 194, p<0.01
**Factor 3 – Prestige**	1.77	1.92	1.59	1.58	F = 0.70, df = 194, p=0.55
**Factor 4 – Hospital Orientation**	2.39^c,d^	2.63^c,d^	4.25^a,b^	4.07^a,b^	F = 29.95, df = 194, p<0.01
**Factor 5 – Role Model**	4.27	4.24	4.19	4.83	F = 2.36, df = 194, p=0.07
**Factor 6 – Varied Scope of Practice**	2.85	3.00	2.81	2.78	F = 0.11, df = 194, p=0.95
**Item C – Good match to this career**	2.12	2.05	2.63	2.56	F = 0.77, df = 194, p=0.51
**Item D – Interesting patient population**	4.39	4.48	4.63	4.56	F = 0.55, df = 194, p=0.65
**Item H – Focus on non-urgent care**	3.09^c,d^	3.24^c,d^	2.25^a,b^	1.33^a,b^	F = 9.55, df = 194, p<0.01
**Item O – Dislike for uncertainty**	1.95	1.86	1.50	1.67	F = 0.73, df = 194, p=0.54
**Item P – Prefer medical to social problems**	1.79	2.19	2.50	2.22	F = 2.57, df = 194, p=0.06
**Item R – Research interest**	1.66^c^	2.10^c^	3.00^a,b,d^	1.78^c^	F = 5.11, df = 194, p<0.01
**Item AA - Short postgraduate training**	2.62^d^	2.46	1.75	1.66^a^	F = 2.85, df = 194, p=0.04

NB. ^a,b,c,d^ indicates the groups from which the group in question differs according to LSD post-Hoc test
